# Evaluating an RNA-based test for proliferation assessment and recurrence prediction in early HR+/HER2− breast cancer

**DOI:** 10.3389/pore.2025.1612113

**Published:** 2026-07-08

**Authors:** Marcus Vetter, Elena Diana Chiru, Anna Gasior, Joanna Gorniak, Sara Rollinson, Leanne Gough, Mathew Harrison, Martina Sonderegger-Stalder, Matthias Matter, Simone Muenst, Christian Kurzeder

**Affiliations:** 1 Center for Oncology and Hematology, Medical University Clinic, Cantonal Hospital Basel-Land, Liestal, Switzerland; 2 Medical Faculty, Basel University, Basel, Switzerland; 3 APIS Assay Technologies Ltd., Manchester, United Kingdom; 4 Institute of Medical Genetics and Pathology, University Hospital Basel, Basel, Switzerland; 5 Breast Care Center, University Hospital Basel, Basel, Switzerland

**Keywords:** breast cancer, recurrence score, molecular subtyping, recurrence risk, proliferation, Oncotype DX, Prosigna PAM50, Ki67

## Abstract

Multigene signatures like Oncotype DX and Prosigna Prediction Analysis of Microarray 50 (PAM50) help estimate distant recurrence risk in patients with luminal breast cancer receiving endocrine therapy. Determining the benefit of adjuvant chemotherapy, tests are costly and not always supported through reimbursement. We aimed to assess the utility of the APIS Breast cancer subtyping kit (BCSK) and its proliferation score (PS), as a potential prognostic assay. We analysed 141 adult patients with early luminal HER2- breast cancer diagnosed between 2020 and 2022 at Cantonal Hospital Basel-Land and Basel University Hospital. All patients had a valid OncotypeDX^®^ Recurrence Score (RS) and received at least one line of adjuvant therapy. Molecular subtype and PS were obtained using the APIS BCSK. We performed the Prosigna PAM50 risk of recurrence (ROR) test on a subset of 59 patients. Our findings showed high concordance at the single marker level (estrogen receptor -ESR1, progesterone receptor - PGR, human epidermal growth factor receptor 2 - ERBB2 and marker of proliferation Ki-67 - MKI67), and between the BCSK and PAM50 subtype (overall percent agreement, OPA-71.2%). Notably, BCSK showed stronger agreement with immunohistochemistry (IHC)-based subtypes (OPA: 71.2%) than PAM50 with IHC (OPA: 54.2%). The BCSK PS correlated moderately with Prosigna PAM50 ROR (ρ = 0.4787) and more weakly with Oncotype DX RS (ρ = 0.4006). The correlation between ROR and RS was weak (ρ = 0.2255). In this preliminary comparison, the APIS BCSK and PS demonstrate promising potential as an initial molecular subtyping and risk stratification tool for breast cancer patients.

## Introduction

Evaluation of proliferation markers in breast cancer is crucial for assessing prognosis and guiding therapy. Standard diagnostic methodologies include histological grading and Ki67 measurement. Together with the breast cancer subtypes, characterized by estrogen receptor (ER), progesterone receptor (PR), and human epidermal growth factor receptor 2 (HER2) status, these carry significant prognostic and therapeutic implications [1]. Approximately 65% of breast cancer patients have ER+ and/or PR+/HER2- tumors [2]. While adjuvant chemotherapy and endocrine treatments led to improved survival outcomes for these patients, a substantial proportion derive minimal benefits from adjuvant chemotherapy [3]. It is essential to identify high-risk patients who benefit from addition of chemotherapy, and patients with lower recurrence risk in whom unnecessary chemotoxicity and financial burden can be spared. This challenge has led to the emergence of multigene signatures, aimed at evaluating the risk of recurrence and guiding the use of adjuvant therapy [4, 5]. Despite the clinical validation of several multigene assays for recurrence risk assessment, baseline proliferation assessment still relies on Ki67 immunohistochemistry (IHC), a method prone to preanalytical and analytical variability. Together with interobserver variability, this leads to limited reliability and reproducibility, which can impact clinical decision making [6, 7].

Clinically validated multigene prognostic tests, including Prosigna [8], OncotypeDx [9], and Mammaprint [10], play a crucial role in breast cancer management. These tests are instrumental in providing prognostic information to guide treatment decisions. All tests incorporate markers of proliferation, emphasizing its significance for breast cancer prognosis. Transcriptome analysis has shown that proliferation integrates a substantial portion of the prognostic information within breast cancer and highlights the pivotal role of proliferation markers within multigene prognostic tests [4, 5].

The aim of this analysis is to compare the proliferative signature (PS) of the newly developed APIS Breast Cancer Subtyping Kit (BCSK) [11] with two clinically validated molecular assays for risk of recurrence, OncotypeDX recurrence score (RS) and Prosigna PAM50 risk of recurrence (ROR). We assessed concordance between signatures, and between signatures and IHC subtypes, and evaluated the potential prognostic value of the APIS BCSK in ER+/HER2- early breast cancer patients.

## Materials and methods

### Patient population

We studied 141 adult patients diagnosed with early luminal HER2- breast cancer between 2020 and 2022 at Cantonal Hospital Basel-Land and Basel University Hospital [12]. All patients had an OncotypeDX^®^ RS score on file and were planned to receive at least one line of adjuvant therapy. Comprehensive clinical and pathological data were collected, including age, tumor stage, tumor grading, nodal status, hormone receptor status, Ki67 expression, and histologic subtype at the time of diagnosis. Treatment details, encompassing the type of surgery and actually received adjuvant therapy regimen, were also documented. Longitudinal follow-up data, including dates and disease-specific recurrence status, were available for the entire study cohort. Because neoadjuvant treatment could include changes between core needle biopsy (CNB) and surgical resection (RES), patients receiving neoadjuvant treatment were excluded from the analysis. The study was approved by the Ethics Committee Nord West Schweiz (www.eknz.ch, proposal number 2023-00108).

### Analysis

Formalin-fixed paraffin-embedded (FFPE) sections were used for RNA extraction using the QIAGEN DSP RNeasy FFPE kit (Qiagen GmBH cat no 73604), and subsequent analysis by the APIS Breast Cancer Subtyping Kit (BCSK), a reverse transcription quantitative polymerase chain reaction (RT-qPCR) based assay detecting expression of *ESR1*, *PGR*, *ERBB2*, *MKI67*, *CCNA2*, *PCNA*, and *KIF23*, as per the instructions for use [13].

OncotypeDX RS was routinely obtained from resection tissue submitted to Exact Sciences, Genomic Health, Redwood City, CA. Results were retrieved from the original patient report and classified as per the manufacturer’s recommendations. For patients over 50 years old, RS 26 and above were considered high risk. For patients below age 50, RS between 21 and 25 were considered medium risk, and 26 and above as high risk. All other patients were defined as low risk as described in the Oncotype DX interpretation guide and per the results of the TAILORx trial [14]. Low risk scores suggest that chemotherapy may be avoided while intermediate and high-risk scores suggest that chemotherapy may be considered for patient treatment.

The BCSK uses relative PCR expression of 4 proliferation markers (MKI67, CCNA2, PCNA2 and KIF23) applying a logistic model to calculate a proliferation score (PS) between 0 and 1 with a value below 0.5 reported as low proliferation and greater than 0.5 as high proliferation [11].

In a subset of 59 patients the Prosigna PAM50 intrinsic subtype and ROR were assessed using the PAM50 Codeset following the NanoString protocol on the nCounter platform (NanoString Technologies, Seattle, WA) by a central laboratory (Propath UK Limited, Willow Court, Netherwood Road, Hereford, HR2 6JU, United Kingdom) [8] in addition to the assessment described above. Subtype calls were categorized into luminal A, luminal B, HER2-enriched, or basal-like based on the Prosigna PAM50 analysis results. Prosigna ROR scores ≤40 were considered low risk, ROR scores 41–60 were considered intermediate risk, and ROR scores >60 were designated as high risk.

Eligibility of patients for the study cohort was based on the IHC (ER, PR, HER2 and Ki67) score of the core needle biopsies performed at the pathology institutes at Basel University and Cantonal Hospital Baselland [15]. For the 59 patient samples used for PAM50 assessment, the Ki67 staining was repeated on the resection specimen as it could have changed from the original assessment. IHC on resected formalin-fixed paraffin-embedded tissue was carried out by Source BioScience (1 Orchard Place, Nottingham Business Park, Nottingham, NG8 6PX) because of limited capacity at the pathology institutes at Basel University and Cantonal Hospital Baselland. IHC was also repeated for ER, PR and HER2 in those samples which were labelled by the PAM50 algorithm as basal or HER2+. All staining was carried out using the Ventana Ultra Benchmark using the Ventana Ultra Assays for ER (Clone SP1), PR (Clone 1E2), Ki67 (Clone 30/9) and HER2 (Clone 4B5) following the manufacturer’s instructions for each antibody. Slides were scored by as per the ASCO CAP guidelines [16, 17]. Ki67 status was considered positive above 20% cell staining following the local practice standard. IHC molecular subtype was defined per the European Society for Medical Oncology (ESMO) guidelines [18].

### Statistical methods

Spearman’s rank correlation was used to study associations between continuous variables. R^2^ values were generated using linear line of fit. Pearsons Chi [2] tests were used to assess the goodness of between recurrence scores. Two tailed up-paired T-tests, assuming equal variance were used to compare cohorts. Median values, Quartiles (25th and 75th percentiles) and interquartile ranges were calculated to compare data distributions. Positive percent agreement (PPA), negative percent agreement (NPA) and overall percent agreement (OPA) were calculated to compare concordance between methods. Assuming a 30% baseline proportion this study has 80% power at a 0.05 significance level to detect an 18% change in proportion. For the PAM50 cohort this equates as sufficient power to detect 11 samples changing risk call between tests.

All statistical analyses were performed with Jmp (V16.1.0) (SAS Institute Inc).

## Results

Clinical data and OncotypeDX risk scores (RS) were available for 141 patients, PAM50 was tested in a subgroup of 59 patients. All patients enrolled in the study presented with luminal A/B HER2- tumors, as determined by standard IHC on core needle biopsy. The mean age of the patients was 59 years (age range 29–83 years), with similar distribution in the PAM50 subset (mean 63 years, range 30–80, p = 0.36). Nodal status was similar in the entire cohort and the PAM50 subset (p = 0.39). Most patients had limited lymph node involvement, 58.8% of patients had N0 disease in the full cohort (81/141) and 62.7% (37/59) in the PAM50 subset. Most patients had stage II or lower disease, endocrine therapy was administered to 73.1% of patients in the full cohort and 64.6% in the PAM50 subset. The remaining patients received combined endocrine and chemotherapy, 22.0% in the full cohort and 28.8% in the PAM50 subset. Seven patients did not receive adjuvant treatment due to refused treatment or a multidisciplinary team decision. Full demography is provided in Table 1.

**TABLE 1:** Demographics and tumor characteristics.

	Description	Number (%) in whole cohort	Number (%) in the PAM50 cohort	P value
Age	≤50	36 (31.4%)	12 (20.3%)	0.36
	>50	105 (68.6%)	47 (79.7%)	
Age	Median (range)	59 (29–83)	63 (30–80)	
Histological subtype	Lobular	19 (13.5%)	4 (6.8%)	0.28
	Ductal	114 (80.9%)	51 (86.4%)	
	Mixed	8 (5.1%)	4 (6.8%)	
BMI	1 = <18.5	2 (1.5%)	1 (1.7%)	0.91
	2 = 18.5–24.9	77 (56.9%)	33 (58.9%)	
	3 = 25–29.9	34 (25.6%)	16 (28.6%)	
	4 = 30–34.9	16 (12.0%)	4 (7.1%)	
	5 = 35–39.9	3 (2.3%)	1 (1.8%)	
	6 = >40	1 (0.8%)	1 (1.8%)	
	Not known	8	3	
IHC	​	Median % (Range %) in whole population	Median % (Range %) in the PAM50 Subset	P-value
ER IHC (% staining)	Median (range)	100% (75%–100%)	100% (75%–100%)	
PR IHC (% staining)	Median (range)	75% (0%–100%)	60% (0%–100%)	
KI67 IHC (% staining)	Median (range)	20% (0%–60%)	25% (0%–50%)	
IHC-based subtype^a^	​	Number (%) in the whole cohort	Numer (%) in the PAM50 subset	P-value
	Luminal A	76 (53.9%)	28 (47.5%)	0.41
	Luminal B	65 (46.1%)	31 (52.5%)	
Tumour size (mm)	Median (range)	21 mm (6–120 mm)	24 mm (9–120 mm)	
Nodal status (pN)	0	81 (58.3%)	37 (62.7%)	0.82
	1	56 (40.3%)	21 (35.6%)	
	2	-	-	
	3	2 (1.4%)	1 (1.7%)	
	Not known	2 (1.4%)	-	
Grade	1	14 (9.9%)	2 (3.6%)	0.18
	2	75 (53.2%)	30 (50.8%)	
	3	50 (35.5%)	27 (45.8%)	
	Not known	4 (2.6%)	-	
Stage (UICC)	IA	44 (31.4%)	15 (25.4%)	0.95
	IB	1 (0.7%)	-	
	IIA	54 (38.6%)	24 (40.7%)	
	IIB	32 (22.9%)	17 (28.8%)	
	IIIA	4 (2.9%)	1 (1.7%)	
	IIIB	2 (1.4%)	1 (1.7%)	
	IIIC	2 (1.4%)	1 (1.7%)	
	IV	1 (0.7%)	-	
Oncotype DX RS	Median (range)	18 (0–53)	20 (0–53)	
Adjuvant therapy	None	7 (5.0%)	4 (6.8%)	0.47
	Endocrine therapy	103 (73.1%)	38 (64.4%)	
	Combined Endocrine and Chemotherapy	31 (22.0%)	17 (28.8%)	
Chemotherapy	Adjuvant therapy	31 (22.0%)	17 (29.8%)	

^a^ IHC based subtype based on KI67 results from CNB as per enrolment into the study cohort.

BMI, body mass index; IHC, immunohistochemistry; mm, millimeter; RS, the Oncotype DX Breast Recurrence Score; UICC, Union of International Cancer Control.

### Comparison of subtypes by IHC and BCSK

Individual gene expression analysis using the APIS BCSK revealed a high level of concordance with IHC results. Full concordance was observed for ESR1/ER and ERBB2/HER2 and high concordance was observed for progesterone receptor PGR/PR (86.52%) and Ki67/MKI67 from resected specimens. Ki67 from IHC scoring from the CNB samples showed a lower concordance with RT-qPCR results (PPA 43.55%), possibly due to known heterogeneity within tumours [19]. Repeat IHC from the resection sample for ER, PR and HER2 matched results from the core needle biopsy (CNB) sample (N = 4). The substantial level of overall concordance with Ki67 IHC results of 71.19%, supports the utility of the BCSK in assessing proliferation within this dataset (Table 2). Ki67 was also assessed using suggested Ki67 international working group thresholds (0%–5%, 6%–29%, >30%) [7]. All comparisons showed statistical difference (0–5 vs. 6–29: p = 0.0057; 6–29 vs. 30: p = 0.0120; 0–5 vs. 30: p = 0.0002). A comparison of biomarker assessment was also performed as continuous variables (% staining versus delta Ct (dCt)) showing a correlation of 0.1663 for ER (p = 0.0136), 0.6717 for PR (p < 0.0001), 0.4445 for Ki67 CNB (p < 0.0001) and 0.7005 for KI67 resected tissue (RES) (p < 0.0001). No comparison could be made for HER2 as all values were negative and no % staining was available.

**TABLE 2:** Concordance between IHC and BCSK in the study cohort for individual marker calling.

	PPA (% (95% CI), N/Total)	NPA (% (95% CI), N/Total)	OPA (% (95% CI), N/Total)
ER/ESR1	99.29% (96.11–99.98) 140/141	NA	99.29% (96.11–99.98) 140/141
PR/PGR	86.52% 79.76–91.69) 113/129	75.00% (42.81–94.51) 9/12	86.52% (79.76–91.69) 122/141
HER2/ERBB2	NA	100.00% (97.42–100) 141/141	100.00% (97.42–100) 141/141
Ki67/MKI67^a^	43.55% (30.99–56.74) 27/62	84.81% (74.97–91.90) 67/79	66.67% (58.24–74.37) 94/141
Ki67/MKI67#	71.19% (54.48–86.70) 24/33	69.23% (48.21–85.67) 18/26	71.19% (57.92–82.24) 42/59

^a^ Concordance versus Ki67 IHC results generated on CNB material and RT-qPCR on resected tissue, # concordance versus IHC and RT-qPCR on matched resected tissue.

CI, confidence interval; ER/ESR1, estrogen receptor; HER2/ERBB2, human epidermal growth factor receptor 2; Ki67/MKI67, marker of proliferation Ki67; N, number of samples; NPA, negative percent agreement; OPA, overall percent agreement; PPA, positive percent agreement.

### Comparison of subtype calling between IHC, BSCK and PAM50

A comparative analysis of luminal A/B subtype calls was conducted between the different assays (supplementary data Table 1). The concordance between IHC and the BCSK was 71.2% and aligns with previously published data [11]. We observed moderate concordance between IHC and PAM50 (54.2%). A higher concordance was found between the BCSK and PAM50 (71.2%). The individual subtype and PCR data are included within the Supplementary Table S2.

**FIGURE 1:**
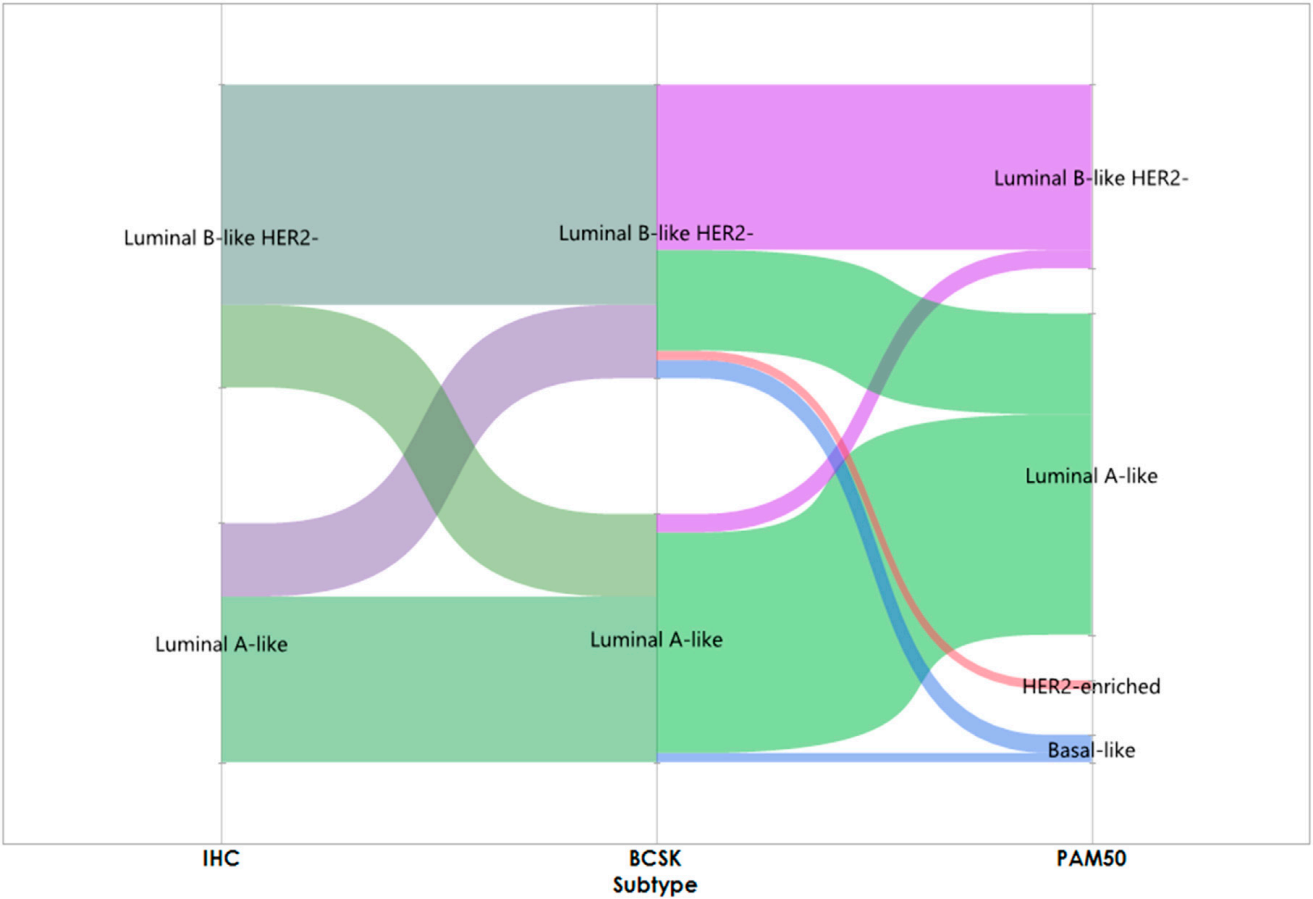
Sankey plot showing the relationship between subtypes as called by Immunohistochemistry (IHC), APIS Breast Cancer Subtyping Kit (APIS BCSK) and Prosigna PAM50 (PAM50).

Cases identified as luminal A by IHC (N = 26) were also predominantly classified as luminal A by both PAM50 and BCSK. However, 7 cases were categorised as luminal B HER- by PAM50, and 8 by the BCSK. Cases identified as luminal B HER2- by IHC (N = 33) had lower concordance and only 13 cases were consistently classified as luminal B HER2- by both PAM50 and BCSK. BCSK classified 24/34 cases as luminal B HER2- and 9 cases classified as luminal A. PAM50 classified 13/34 cases as luminal B HER2- and 16 cases were classified as luminal A. Eight cases were classified as luminal A by BCSK and PAM50. Notably, three cases initially classified as IHC Luminal B HER2- were reclassified as Basal-like, and one case was classified as HER2-enriched by PAM50. A Sankey plot depicting the relationship between the subtype called by each of the methods is shown in Figure 1.

### Relationship between APIS BCSK proliferation score (PS), oncotype DX RS, and prosigna PAM50 ROR

**FIGURE 2:**
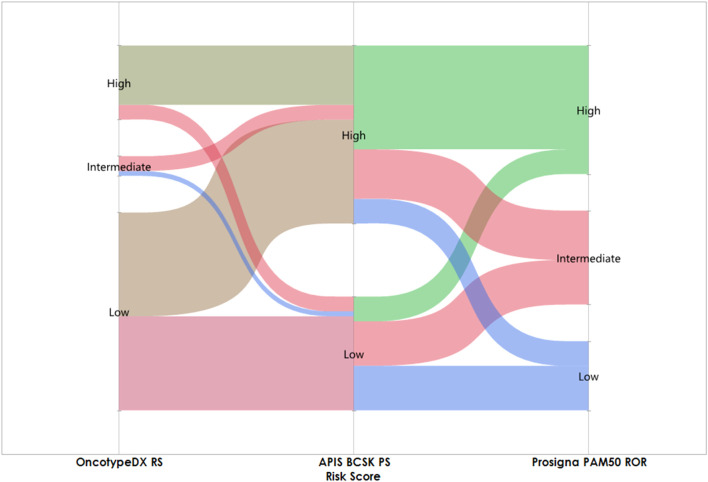
Sankey plot showing the relationship between risk scores as called by OncotypeDX (OncotypeDX RS), APIS Breast Cancer Subtyping Kit (APIS BCSK PS) and Prosigna PAM50 (Prosigna PAM50).

Median and interquartile ranges were measured between the full cohort and the PAM50 case subset lending support to the validity of the analysis for both the OncotypeDX Recurrence Score (RS) and APIS Proliferation Score (PS) (Supplementary Table S3). The Prosigna ROR classified 14 (23.7%) cases low risk, 26 (44.1%) intermediate and 19 (32.3%) high risk. According to OncotypeDX RS 40 (67.8%) cases were low risk, 4 (6.8%) medium and 15 (25.4%) high risk. The APIS proliferation score classified 23 (39.0%) cases as low risk, and 36 (61.0%) as high risk. A Sankey plot depicting the relationship between the risk called by each of the methods is shown in Figure 2. A comparison of signature performance was made by treating Prosigna ROR, OncotypeDX RS and APIS PS as continuous variables. A strong positive correlation was observed between the APIS PS and Prosigna ROR (ρ = 0.4787) and a moderate correlation between APIS PS and OncotypeDX RS (ρ = 0.4006). The findings indicate that higher APIS PS tends to positively correlate with higher OncotypeDX RS, though the relationship is not as strong as with Prosigna ROR. Little correlation was observed between the OncotypeDX RS and Prosigna ROR in this cohort (ρ = 0.2255). Within our study time frame, four patients experienced clinical relapse, of which 2 were classified as high risk by both the RS and PS and one case was classified as high risk by the Prosigna ROR score. For individual patient scores and correlations see Supplementary Figure S1.

The APIS BCSK PS was compared with OncotypeDX RS and Prosigna ROR at the patient level. Since both the Prosigna ROR and OncotypeDX RS have an intermediate or medium risk group overall percentage agreement (OPA) was calculated by alternatively labelling the intermediate group as either low or high risk (Supplementary Table S5). Indeed, the low correlation between the APIS BCSK and OncotypeDX RS is supported by the comparison of the Prosigna ROR and OncotypeDX RS scores, with an OPA of 57.6% when calling intermediate cases as low risk and 45.8% when calling the same cases as high risk. The lower OPA when classifying intermediate cases as high risk suggests a discrepancy in the risk classification between Prosigna ROR and OncotypeDX RS for these intermediate risk cases.

Chemotherapy treatment decisions were guided by OncotypeDX with combined discussion at the multidisciplinary team meeting, in the PAM50 cohort 4 cases were not assigned adjuvant therapy, 4 patients with a low OncotypeDX RS received adjuvant chemotherapy in addition to hormone therapy and 6 patients with a high score received hormone therapy alone. A breakdown of therapy as would have been assigned by APIS PS, Prosigna PAM50 and Ki76 IHC are shown in Supplementary Table S4.

## Discussion

IHC is the gold standard for molecular subtyping in breast cancer. Because of challenges regarding accuracy and reproducibility, especially of the Ki67 proliferation marker, diagnostic assays have been developed to measure prognostic markers at the mRNA level. The APIS BCSK achieves high concordance with traditional IHC methods [11]. The individual marker analysis presented here further validates the assay, particularly with regards to its accuracy in determining hormone receptor and proliferation status and confirms the potential of the BCSK as a reliable tool for molecular profiling in breast cancer patients [11].

In early ER+/HER2- breast cancer most patients with intermediate clinical risk receiving 5 years of adjuvant endocrine therapy alone do not experience recurrence [20]. Multigene gene expression assays have been developed to better identify patients in whom additional adjuvant chemotherapy could be omitted [21]. The assays heavily rely on proliferation assessment, where co-regulation of proliferation markers, as suggested by Venet et al. [4], implies a potential interchangeability in their utilization. The inclusion of a proliferation signature in the BCSK offers an opportunity for comprehensive and integrated molecular profiling at the place and time of diagnosis. The proliferation signature within the APIS BCSK test might be used for decisions about further multigene testing and recommendation of treatment escalation. The proliferation signature can address analytical issues with KI67 and its kinetics after neo-adjuvant chemotherapy [22].

Here we present the first comparative data between the APIS BCSK and Prosigna PAM50 molecular subtype assay. The concordance analysis highlights the significance of Ki67 status in ER+/HER2- breast cancer and underscores the superior performance of molecular methods, with the APIS BCSK and Prosigna PAM50 achieving the highest concordance. The observed influence of *ESR1* and *PGR* expression on risk of recurrence emphasizes the importance of considering hormone receptor status when interpreting results from different risk assessment tools. Higher expression of ER is linked to a lower risk of recurrence since the marker is a druggable target. PR expression is upregulated by ER, and can modulate ER action, therefore higher expression of PR is also associated with better prognosis. Clinically PR negativity in ER+ tumors confers a higher risk of recurrence [23], predisposing to development of endocrine resistance. Tumors with ER+/PR- tumor gene expression profiles are more like the luminal B subtype and have poorer prognosis [1, 24, 25]. To determine the adjuvant treatment strategy multigene testing is only recommended for patients with early-stage luminal A or -B breast cancer. A routine proliferation signature from the initial core needle biopsy tissue within the APIS BCSK offers a streamlined approach, potentially revolutionizing the diagnostic landscape for breast cancer and leading to more cost-effective therapy decisions.

Our initial assessment of the APIS BCSK PS aimed to explore its potential as a tool for supporting adjuvant treatment decisions. Our study showed a positive correlation between APIS PS and the already established recurrence risk scores Prosigna ROR and OncotypeDX RS and demonstrated that higher proliferation markers are linked to an increased risk of recurrence. We also observed a strong correlation between the APIS BCSK PS and the Prosigna PAM50 ROR and a weaker correlation with the OncotypeDX RS.

As previously reported in the literature there is a variability in the risk categorization between the different methods, highlighting the diverse nature of these risk assessment tools [26]. While the intermediate risk category was predominant in the Prosigna ROR classification, most cases fell into the low-risk RS category. The APIS PS appears to categorize a significant portion of cases as high risk suggesting a more conservative approach to risk stratification. A weak correlation was observed between the Prosigna ROR and OncotypeDX RS in this cohort. Similar findings have been described in the OPTIMA study, in which fewer than 40% of all patients were classified alike by ROR and RS [26]. Despite of this, validation studies for both Prosigna ROR and OncotypeDX RS assays support successful in reporting of genomic risk of disease recurrence [25]. Proliferative signatures drive the scores of most prognostic tests except for the OncotypeDX test, where proliferation is thresholded and the scoring model is also driven by the estrogen signaling expression module, including *PGR* expression [5], [27]. Long term clinical validation show that the assays perform slightly differently, with Prosigna ROR providing more prognostic information in endocrine treated node negative patients than OncotypeDX RS [24]. The data also suggests the ROR contains molecular components that are more specifically prognostic for late recurrence than OncotypeDX RS [25].

In this preliminary comparison, the APIS BCSK and PS demonstrate promising potential as an initial molecular subtyping test for breast cancer patients. While the full clinical validation of its prognostic performance requires further testing, its ability to correctly identify molecular subtypes meets a more immediate clinical need. The APIS BCSK’s potential to serve as an accessible and cost-effective alternative (APIS test being cost neutral to IHC versus the cost of prognostic gene expression assays ranging from £1500 to £2500) is particularly relevant for patients who may not meet the formal criteria for reimbursement of one of the currently established molecular prognostic tests. Given that the APIS BCSK results can be reported from core needle biopsy at the initial diagnosis, it has the potential to play a pivotal role in informing early diagnostic and treatment decisions. One crucial aspect to consider is the decision-making process for ordering additional tests such as Prosigna PAM50 or OncotypeDX. The APIS BCSK has the potential to serve as a valuable standalone test for patients with very high or low APIS PS scores especially when cost constraints or reimbursement eligibility are limiting factors. In cases where the APIS BCSK reveals a moderate PS, close to the cutoff, careful consideration of all molecular results is advised before ordering additional tests.

This study is limited by its small cohort size, which may not fully represent the heterogeneity of the broader patient population, thereby constraining the generalizability of the findings. Additionally, the limited duration of follow-up and low number of clinical events further restrict the strength of outcome-related conclusions. The subset used for comparison between PAM50 and OncotypeDX assays was even smaller, which may reduce the reliability of this specific analysis.

Furthermore, most immunohistochemistry data were derived from core needle biopsy specimens, with limited validation on corresponding resection samples which was only performed for the discordant PAM50 cases. Although ER, PR, and HER2 showed reproducibility between CNB and resection, Ki67 was notably less consistent - likely due to both pre-analytical variables and biological heterogeneity between smaller biopsies and larger resections [28]. Although misclassifications were observed, IHC was treated as the truth. The Prosigna PAM50 and APIS BCSK subtype were not performed at the time of diagnosis rather retrospectively and therefore could not be part of the discussion at the multidisciplinary team determining the treatment.

In the future, results from the APIS BCSK being made available for the multidisciplinary team discussion could help improve treatment and further diagnostic decisions for patients. Future studies with larger sample sizes and extended follow-up are warranted to validate these findings. Such studies may also enable assessment of the number of BCSK tests required per omitted adjuvant chemotherapy, stratified by tumor characteristics.

In conclusion, while our initial findings are promising, further validation studies are warranted to support the clinical utility and reliability of the APIS BCSK PS. Fully validated, this locally available and cost-effective test could mark significant progress in adjuvant treatment decision support for luminal breast cancer patients.

## Data Availability

The original contributions presented in the study are included in the article/Supplementary Material, further inquiries can be directed to the corresponding author.
