# Supplementary material for: Evaluating an RNA-based test for proliferation assessment and recurrence prediction in early HR+/HER2− breast cancer
Source: Pathol Oncol Res. 2026 Jul 8;31:1612113. doi: 10.3389/pore.2025.1612113 (PMC13388230; doi:10.3389/pore.2025.1612113)
Supplement: Supplementary file 1 [file Supplementaryfile1.docx]

**Supplementary information**

**Table S1. Subtypes for the study subjects as determined by IHC, APIS BCSK and Prosigna PAM50 subtype call.**

| **IHC call*** | **BCSK** | **N (%)** |
| --- | --- | --- |
| Luminal A | Luminal A | 18 (30.5%) |
|  | Luminal B Her2 - | 8 (13.6%) |
| Luminal B Her2 - | Luminal A | 9 (15.3%) |
|  | Luminal B Her2 - | 24 (40.7%) |
| **IHC call*** | **PAM50** |  |
| Luminal A | Luminal A | 19 (32.2%) |
|  | Luminal B Her2 - | 7 (11.9%) |
| Luminal B Her2 - | Luminal A | 16 (27.1%) |
|  | Luminal B Her2 - | 13 (22.0%) |
|  | Basal like | 3 (5.1) |
|  | HER2 Enriched | 1 (1.7%) |
| **PAM50** | **BCSK** |  |
| Luminal A | Luminal A | 24 (40.7%) |
|  | Luminal B Her2 - | 11 (18.6%) |
| Luminal B Her2 - | Luminal A | 2 (3.4%) |
|  | Luminal B Her2 - | 18 (30.5%) |
| Basal like | Luminal A | 1 (1.7%) |
| Basal like | Luminal B Her2 - | 2 (3.4%) |
| HER2 enriched | Luminal B Her2 - | 1 (1.7%) |

*IHC data as derived from resection specimens used as input for the BCSK and Prosigna assay

**Table S2. Delta Ct (dCt) reverse transcription quantitative polymerase c chain reaction (RT-qPCR) values, subtype calls for immunohistochemistry (IHC), APIS Breast Cancer Subtyping Kit (BCSK), Prosigna PAM50 reoccurrence of risk (ROR), OncotypeDX Risk Score (RS) and APIS Proliferation calls for the data set.**

|  |  | BCSK target calls and subtypes | | | | | | | PAM50 data | | | Oncotype data | |
| --- | --- | --- | --- | --- | --- | --- | --- | --- | --- | --- | --- | --- | --- |
| APIS_ID | IHC subtype | dCt ESR1 | dCt PGR | dCt ERBB2 | dCt MKI67 | BC subtype | Proliferation Score | Proliferation call | PAM50 Subtype | PAM50 ROR | PAM50 Risk | Oncotype RS | Oncotype risk |
| C223367 | Luminal B-like HER2- | -0.15 | -0.72 | -1.24 | -1.56 | Luminal A-like | 0.412 | 0 | Basal-like | 48 | Intermediate | 36 | High |
| C223340 | Luminal B-like HER2- | 1.59 | 2.79 | -2.48 | -0.58 | Luminal B-like HER2- | 0.477 | 0 | Basal-Like | 49 | Intermediate | 20 | Low |
| C223301 | Luminal B-like HER2- | 0.61 | -0.02 | -0.86 | 0.83 | Luminal B-like HER2- | 0.898 | 1 | Basal-like | 61 | High | 50 | High |
| C223329 | Luminal B-like HER2- | 2.33 | -2.88 | 0.25 | 0.50 | Luminal B-like HER2- | 0.881 | 1 | HER2-enriched | 86 | High | 53 | High |
| C224323 | Luminal B-like HER2- | -1.91 | -2.28 | -0.64 | -1.55 | Luminal A-like | 0.268 | 0 | Luminal A-like | 38 | Intermediate | 35 | High |
| C223325 | Luminal B-like HER2- | 0.62 | 4.41 | -0.46 | -0.06 | Luminal B-like HER2- | 0.624 | 1 | Luminal A-like | 43 | Intermediate | 30 | High |
| C223373 | Luminal B-like HER2- | 0.00 | 3.08 | -0.45 | -0.14 | Luminal B-like HER2- | 0.802 | 1 | Luminal A-like | 54 | Intermediate | 23 | Medium |
| C223282 | Luminal A-like | 3.08 | 1.35 | 0.29 | -0.68 | Luminal A-like | 0.521 | 1 | Luminal A-like | 55 | High | 17 | Low |
| C224309 | Luminal A-like | -1.25 | -1.88 | -0.85 | -3.05 | Luminal A-like | 0.032 | 0 | Luminal A-like | 26 | Intermediate | 32 | High |
| C224311 | Luminal B-like HER2- | 1.01 | 2.76 | 0.05 | -0.47 | Luminal B-like HER2- | 0.579 | 1 | Luminal A-like | 36 | Intermediate | 18 | Low |
| C223363 | Luminal B-like HER2- | 0.68 | 2.34 | 0.14 | -0.40 | Luminal B-like HER2- | 0.811 | 1 | Luminal A-like | 28 | Low | 23 | Medium |
| C224347 | Luminal A-like | 3.74 | -1.23 | -0.46 | 0.88 | Luminal B-like HER2- | 0.945 | 1 | Luminal A-like | 26 | Low | 49 | High |
| C223307 | Luminal B-like HER2- | 3.52 | 3.94 | -0.81 | -0.24 | Luminal B-like HER2- | 0.795 | 1 | Luminal A-like | 55 | Intermediate | 6 | Low |
| C223356 | Luminal B-like HER2- | 1.57 | 0.86 | -1.90 | -0.68 | Luminal A-like | 0.597 | 1 | Luminal A-like | 23 | Intermediate | 24 | Low |
| C224320 | Luminal B-like HER2- | 0.47 | 4.21 | 0.24 | -0.28 | Luminal B-like HER2- | 0.661 | 1 | Luminal A-like | 35 | Low | 19 | Low |
| C224318 | Luminal A-like | 1.88 | 2.04 | -2.41 | -2.11 | Luminal A-like | 0.240 | 0 | Luminal A-like | 32 | Intermediate | 16 | Low |
| C223386 | Luminal A-like | 1.52 | 2.65 | -0.68 | -1.68 | Luminal A-like | 0.312 | 0 | Luminal A-like | 26 | Low | 18 | Low |
| C224319 | Luminal A-like | 3.28 | 3.26 | 1.91 | -0.81 | Luminal A-like | 0.498 | 0 | Luminal A-like | 48 | Intermediate | 11 | Low |
| C224341 | Luminal B-like HER2- | 0.52 | 1.33 | -1.06 | -1.09 | Luminal A-like | 0.460 | 0 | Luminal A-like | 30 | Low | 24 | Low |
| C223292 | Luminal A-like | -1.66 | 2.36 | -1.47 | -2.72 | Luminal A-like | 0.110 | 0 | Luminal A-like | 12 | Low | 21 | Medium |
| C224324 | Luminal B-like HER2- | 2.58 | 2.10 | -0.66 | -1.53 | Luminal A-like | 0.374 | 0 | Luminal A-like | 33 | Low | 13 | Low |
| C224307 | Luminal A-like | 1.22 | 1.71 | 0.17 | -0.12 | Luminal B-like HER2- | 0.589 | 1 | Luminal A-like | 55 | High | 24 | Low |
| C224339 | Luminal B-like HER2- | 1.39 | -0.63 | -1.75 | -1.12 | Luminal A-like | 0.548 | 1 | Luminal A-like | 56 | Intermediate | 21 | Low |
| C223290 | Luminal A-like | 2.29 | -2.23 | -1.16 | -0.65 | Luminal A-like | 0.730 | 1 | Luminal A-like | 54 | Intermediate | 38 | High |
| C223384 | Luminal A-like | 2.66 | 4.35 | -2.50 | -2.61 | Luminal A-like | 0.113 | 0 | Luminal A-like | 55 | High | 0 | Low |
| C224315 | Luminal A-like | 1.50 | -0.32 | -0.96 | -1.24 | Luminal A-like | 0.449 | 0 | Luminal A-like | 32 | Low | 21 | Low |
| C223354 | Luminal A-like | -0.51 | 2.30 | -1.37 | -3.53 | Luminal A-like | 0.019 | 0 | Luminal A-like | 20 | Low | 16 | Low |
| C224332 | Luminal A-like | 2.25 | 2.52 | 0.06 | -1.55 | Luminal A-like | 0.222 | 0 | Luminal A-like | 31 | Low | 12 | Low |
| C224338 | Luminal A-like | 2.37 | 1.30 | -0.88 | -1.88 | Luminal A-like | 0.197 | 0 | Luminal A-like | 37 | Intermediate | 14 | Low |
| C223370 | Luminal B-like HER2- | 1.24 | 3.30 | -0.14 | -0.78 | Luminal A-like | 0.625 | 1 | Luminal A-like | 31 | Low | 10 | Low |
| C224316 | Luminal B-like HER2- | 1.59 | -0.23 | -0.64 | -0.79 | Luminal A-like | 0.472 | 0 | Luminal A-like | 52 | High | 20 | Low |
| C224331 | Luminal B-like HER2- | 1.84 | 6.95 | -0.87 | 1.72 | Luminal B-like HER2- | 0.966 | 1 | Luminal A-like | 42 | High | 7 | Low |
| C223285 | Luminal B-like HER2- | 2.73 | 3.02 | -1.09 | -0.13 | Luminal B-like HER2- | 0.799 | 1 | Luminal A-like | 17 | Low | 16 | Low |
| C224346 | Luminal B-like HER2- | 1.99 | -0.67 | -0.44 | -0.65 | Luminal A-like | 0.766 | 1 | Luminal A-like | 50 | Intermediate | 30 | High |
| C223283 | Luminal A-like | 3.01 | 3.09 | -0.66 | -1.91 | Luminal A-like | 0.232 | 0 | Luminal A-like | 39 | Low | 12 | Low |
| C223293 | Luminal A-like | 0.13 | 5.63 | -0.33 | -1.35 | Luminal A-like | 0.258 | 0 | Luminal A-like | 46 | Intermediate | 14 | Low |
| C224326 | Luminal A-like | 1.79 | 1.42 | -0.65 | -0.67 | Luminal A-like | 0.606 | 1 | Luminal A-like | 63 | High | 19 | Low |
| C224305 | Luminal A-like | 0.99 | 1.96 | 0.44 | -0.12 | Luminal B-like HER2- | 0.568 | 1 | Luminal A-like | 48 | Intermediate | 20 | Low |
| C223297 | Luminal A-like | 2.89 | 1.31 | -0.44 | -2.62 | Luminal A-like | 0.127 | 0 | Luminal A-like | 37 | Low | 18 | Low |
| C224317 | Luminal B-like HER2- | 1.86 | 3.45 | -1.78 | 0.69 | Luminal B-like HER2- | 0.742 | 1 | Luminal B-like HER2- | 81 | High | 24 | Low |
| C223366 | Luminal B-like HER2- | 0.26 | 1.85 | -0.14 | 0.43 | Luminal B-like HER2- | 0.869 | 1 | Luminal B-like HER2- | 87 | High | 18 | Low |
| C223308 | Luminal A-like | 1.18 | -1.34 | 0.32 | 0.66 | Luminal B-like HER2- | 0.886 | 1 | Luminal B-like HER2- | 69 | High | 31 | High |
| C223304 | Luminal B-like HER2- | 1.98 | -0.57 | 0.38 | 1.19 | Luminal B-like HER2- | 0.963 | 1 | Luminal B-like HER2- | 88 | High | 43 | High |
| C224333 | Luminal B-like HER2- | 1.22 | 0.42 | -2.11 | 0.09 | Luminal B-like HER2- | 0.738 | 1 | Luminal B-like HER2- | 68 | High | 30 | High |
| C223318 | Luminal B-like HER2- | 2.68 | 0.09 | 0.77 | 0.24 | Luminal B-like HER2- | 0.945 | 1 | Luminal B-like HER2- | 80 | High | 40 | High |
| C223360 | Luminal A-like | 1.23 | 4.33 | -1.17 | -0.23 | Luminal B-like HER2- | 0.634 | 1 | Luminal B-like HER2- | 69 | High | 25 | Medium |
| C223305 | Luminal A-like | 2.74 | -2.94 | -0.36 | -0.63 | Luminal B-like HER2- | 0.482 | 0 | Luminal B-like HER2- | 76 | High | 19 | Low |
| C223380 | Luminal B-like HER2- | 2.21 | 4.06 | 0.03 | -0.40 | Luminal B-like HER2- | 0.489 | 0 | Luminal B-like HER2- | 66 | High | 23 | Low |
| C223385 | Luminal B-like HER2- | 0.93 | 3.54 | 0.42 | -0.51 | Luminal B-like HER2- | 0.610 | 1 | Luminal B-like HER2- | 63 | High | 18 | Low |
| C223348 | Luminal B-like HER2- | 2.57 | 2.06 | -0.22 | -0.26 | Luminal B-like HER2- | 0.779 | 1 | Luminal B-like HER2- | 63 | High | 22 | Low |
| C223351 | Luminal B-like HER2- | 0.06 | 3.27 | -0.06 | -0.58 | Luminal B-like HER2- | 0.648 | 1 | Luminal B-like HER2- | 84 | High | 14 | Low |
| C224340 | Luminal B-like HER2- | 1.66 | -1.32 | 0.05 | -0.25 | Luminal B-like HER2- | 0.660 | 1 | Luminal B-like HER2- | 80 | High | 32 | High |
| C223346 | Luminal B-like HER2- | 1.20 | -1.00 | -0.96 | -0.13 | Luminal B-like HER2- | 0.780 | 1 | Luminal B-like HER2- | 54 | High | 37 | High |
| C223289 | Luminal A-like | -1.55 | -0.63 | -1.86 | -3.39 | Luminal A-like | 0.096 | 0 | Luminal B-like HER2- | 64 | High | 12 | Low |
| C223353 | Luminal A-like | 2.29 | 6.86 | -0.22 | -0.18 | Luminal B-like HER2- | 0.572 | 1 | Luminal B-like HER2- | 60 | Intermediate | 7 | Low |
| C223330 | Luminal B-like HER2- | 3.47 | 2.26 | 0.22 | 0.37 | Luminal B-like HER2- | 0.906 | 1 | Luminal B-like HER2- | 65 | High | 15 | Low |
| C223336 | Luminal B-like HER2- | 4.46 | - | -1.00 | 0.84 | Luminal B-like HER2- | 0.956 | 1 | Luminal B-like HER2- | 71 | High | 21 | Low |
| C223379 | Luminal A-like | 2.54 | 1.86 | -0.77 | -1.54 | Luminal A-like | 0.391 | 0 | Luminal B-like HER2- | 45 | Intermediate | 8 | Low |
| C223322 | Luminal A-like | 3.65 | 5.88 | 0.23 | 0.42 | Luminal B-like HER2- | 0.813 | 1 | Luminal B-like HER2- | 61 | High | 20 | Low |

**Table S3. Quartile values for the molecular scores reported in this study.**

| Cohort | Molecular score | Median | Min | Q1 | Q3 | Max | IQR |
| --- | --- | --- | --- | --- | --- | --- | --- |
| PAM50 subset (N=59) | ROR (0-100) | 52 | 12 | 35 | 64 | 88 | 29 |
|  | RS (0-100) | 20 | 6 | 15 | 30 | 53 | 15 |
|  | Proliferation Score (0-1) | 0.5974 | 0.0189 | 0.3909 | 0.7947 | 0.9660 | 0.4038 |
| Full patient cohort (N=141) | RS (0-100) | 18 | 0 | 12.5 | 24 | 53 | 11.5 |
|  | Proliferation Score (0-1) | 0.4355 | 0.0022 | 0.1617 | 0.6182 | 0.9660 | 0.4565 |

Q1, Q3 (25th and 75th percentiles), IQR (interquartile range).

**Table S4. Assigned adjuvant therapy opposite respective Ki67 IHC, APIS MKI67 RNA, APIS Proliferation Score, Prosigna PAM 50 Risk Score and Oncotype DX Risk Score results.**

| Administered Adjuvant Therapy | Ki67 IHC | | APIS MKI67 | | APIS Proliferation Score | | Prosigna PAM 50 | | | OncotypeDX | | |
| --- | --- | --- | --- | --- | --- | --- | --- | --- | --- | --- | --- | --- |
|  | High | Low | High | Low | High | Low | High | Intermediate | Low | High | Intermediate | Low |
| Chemotherapy | 12 | 5 | 13 | 4 | 14 | 3 | 9 | 6 | 2 | 10 | 3 | 4 |
| No Chemotherapy | 17 | 23 | 18 | 22 | 21 | 19 | 16 | 13 | 11 | 4 | 1 | 35 |

**Table S5 Number of risk score cases according to each assay and overall percentage agreement between the APIS PS, Prosigna ROR, and OncotypeDX RS.**

| **BCSK Proliferation call** | **Prosigna PAM50 ROR** | **N = Proliferation/ROR call** |
| --- | --- | --- |
| Low | Low | 9 |
|  | Intermediate | 9 |
|  | High | 5 |
| High | Low | 5 |
|  | Intermediate | 10 |
|  | High | 21 |
| OPA intermediate/med as low risk (N, %) | | 39 (66.1%) |
| OPA intermediate/med as high risk (N, %) | | 40 (67.8%) |
| BCSK Proliferation call | OncotypeDX RS | N = Proliferation/RS call |
| Low | Low | 19 |
|  | Med | 1 |
|  | High | 3 |
| High | Low | 21 |
|  | Med | 3 |
|  | High | 12 |
| OPA intermediate/med as low risk (N, %) | | 32 (54.2%) |
| OPA intermediate/med as high risk (N, %) | | 34 (57.6%) |
| Prosigna PAM50 ROR | OncotypeDX RS | N = ROR/RS call |
| Low | Low | 11 |
|  | Med | 2 |
|  | High | 1 |
| Intermediate | Low | 12 |
|  | Med | 1 |
|  | High | 6 |
| High | Low | 17 |
|  | Med | 1 |
|  | High | 8 |
| OPA intermediate/med as low risk (N, %) | | 34 (57.3%) |
| OPA intermediate/med as high risk (N, %) | | 27 (45.8%) |

BCSK – APIS Breast Cancer Subtyping Kit, N – number of samples, OPA – Overall percent agreement, APIS PS – Proliferation Score, ROR – PAM50 Risk of Recurrence Score, RS – OncotypeDX Risk Score.

**Supplementary Figure 1. Scatter plots and Spearman’s correlation coefficients (ρ) of the Recurrence Score (RS), Risk of Recurrence (ROR), and Proliferation scores (PS) in this study. Cases with disease specific relapse events are indicated by ◆. The risk score cut-offs for each assay are denoted by dashed lines (for clarity the RS medium risk category in the group of patients under 50 years old has not been plotted since the ≤ 50 and > 50 age groups have been merged for plotting).**
